# Detection of HPV DNA in esophageal cancer specimens from different regions and ethnic groups: a descriptive study

**DOI:** 10.1186/1471-2407-10-19

**Published:** 2010-01-16

**Authors:** Xueqian Wang, Xiuyun Tian, Fangfang Liu, Yiqiang Zhao, Min Sun, Dafang Chen, Changdong Lu, Zhong Wang, Xiaotian Shi, Qingying Zhang, Donghong Zhang, Zhongying Shen, Feng Li, Curtis C Harris, Hong Cai, Yang Ke

**Affiliations:** 1 Key laboratory of Carcinogenesis and Translational Research (Ministry of Education), Peking University School of Oncology, Beijing Cancer Hospital & Institute, 52 Fucheng Rd, Beijing 100142, China; 2 Department of Cell Biology, Health Science Center, Peking University, 38 Xueyuan Rd, Beijing 100191, China; 3 School of Public Health, Health Science Center, Peking University, 38 Xueyuan Rd, Beijing 100191, China; 4 Anyang Cancer Hospital, Anyang, Henan 455000, China; 5 Medical School, Shantou University, 22 Xinling Rd, Shantou, Guangdong 515031, China; 6 Shihezi Medical School, North Second Rd, Shihezi, Xinjiang 832002, China; 7 Laboratory of Human Carcinogenesis, NCI, NIH, 37 Convent Dr. Bethesda, MD, 20892-4255, USA

## Abstract

**Background:**

HPV has been found repeatedly in esophageal carcinoma tissues. However, reported detection rates of HPV DNA in these tumors have varied markedly. Differences in detection methods, sample types, and geographic regions of sample origin have been suggested as potential causes of this discrepancy.

**Methods:**

HPV L1 DNA and HPV genotypes were evaluated in 435 esophageal carcinoma specimens collected from four geographic regions with different ethnicities including Anyang in north China, Shantou in south China, Xinjiang in west China, and the United States. The HPV L1 fragment was detected using SPF1/GP6+ primers. HPV genotyping was performed using genotype specific PCR.

**Results:**

Two hundred and forty four of 435 samples (56.1%) tested positive for HPV L1. Significant differences in detection rate were observed neither among the three areas of China nor between China and the US. HPV6, 16, 18, 26, 45, 56, 57, and 58 were identified in L1 positive samples. HPV16 and 57 were the most common types in all regions, followed by HPV26 and HPV18.

**Conclusions:**

HPV infection is common in esophageal carcinoma independent of region and ethnic group of origin. Findings in this study raise the possibility that HPV is involved in esophageal carcinogenesis. Further investigation with a larger sample size over broader geographic areas may be warranted.

## Background

Esophageal carcinoma is a common malignancy and its mortality rate is among the highest for cancers overall [1]. There is extreme geographic variation in the incidence of esophageal carcinoma, with differences of as much as 300-fold between areas of greatest and least prevalence within a given country, or between one country and another [2]. Substantial alcohol use combined with smoking has been found to greatly increase the risk of esophageal cancer in western countries [3,4]. In contrast, vitamin deficient diets and food containing potential carcinogens have been suggested to be significant risk factors for esophageal cancer in China and other central Asian countries [5-9]. However, the association between esophageal cancer and these identified risk factors is generally weak for the high risk population of China, implying that the major etiology remains to be determined [10,11].

In 1982, Syrjanen found that HPV infection caused pathological lesions in esophageal cancer specimens [12]. Since then many studies which have used a variety of techniques have evaluated the association between HPV and risk of esophageal cancer. However, the results of these studies have been inconsistent, and it has been suggested that this inconsistency may result in part from geographic variation in esophageal cancer prevalence. Most studies that have failed to detect HPV DNA in esophageal tumors were conducted in low-risk areas such as the USA or Europe [13,14]. Studies in high-risk areas have found a significantly higher percentage of HPV in esophageal cancer [15-18].

In this study, we evaluated tissues from 435 esophageal cancers collected from four different regions with multiple ethnicities including Han, Kazakh, American Japanese, American Caucasian, and African American for HPV DNA. We found comparable rates of HPV detection and similar HPV genotypes in the esophageal cancer samples from all of these geographic areas.

## Methods

### Subjects

A total of 435 esophageal cancer samples collected in an unselected manner were used in this study. Of these, 166 were from Anyang, a city in northern China, 103 were from Shantou, a city in southern China, 78 were from Xinjiang, an autonomous region in western China, and 88 were from the United States (US). Among the study subjects, 61.9% were Han, the majority ethnic group of China, 17.9% were Kazakh, a minority group of Chinese living in western China with a high prevalence of esophageal cancer, 9.4% were Caucasian, 9.0% were Japanese, and 1.8% were African-American. The male to female ratio was 2.4:1 (Table 1). The tumor samples included 34 esophageal adenocarcinomas (all from the United States), and 401 esophageal squamous cell carcinomas. The carcinoma tissue specimens were collected from non-necrotic areas of tumor immediately following esophagectomy, and samples were stored at -70°C for subsequent processing. In every case the pathological diagnoses were rendered by local pathologists. Demographic and medical information including age, gender, race, tumor site, tumor stage, family history, and history of smoking and drinking were obtained from patient medical records. Informed consent was obtained from all participants. Institutional review board approval was obtained from all participating institutions.

**Table 1:** Demographic characteristics and TNM stage of subjects by region and HPV infection status

		Region						HPV infection status		
			
		China						Total (N = 435)		
Variables							*P*†			*P*††
		Anyang N = 166 No.(%)	Shantou N = 103 No.(%)	Xinjiang N = 78 No.(%)	Total N = 347 No.(%)	US N = 88 No.(%)		HPV(-) No.(%)	HPV(+) No.(%)	
Age	≤60	87(52.4)	74(71.8)	62(79.5)	223(64.3)	39(44.3)	0.001	121(63.4)	141(57.8)	0.239
	>60	79(47.6)	29(28.2)	16(20.5)	124(35.7)	49(55.7)		70(36.6)	103(42.2)	
										
Gender	Female	55(33.1)	34(33.0)	27(34.6)	116(33.4)	12(13.6)	<0.001	63(33.0)	65(26.6)	0.150
	Male	111(66.9)	69(67.0)	51(65.4)	231(66.6)	76(86.4)		128(67.0)	179(73.4)	
										
Tobacco‡	Never	83(50.6)	54(52.4)	59(75.6)	196(56.8)	19(22.1)	<0.001	104(55.3)	111(45.7)	0.047
	Ever	81(49.4)	49(47.6)	19(24.4)	149(43.2)	67(77.9)		84(44.7)	132(54.3)	
										
Alcohol‡‡	Never	118(72.0)	91(88.3)	65(83.3)	274(79.4)	20(24.4)	<0.001	143(76.5)	151(62.9)	0.003
	Ever	46(28.0)	12(11.7)	13(16.7)	71(20.6)	62(75.6)		44(23.5)	89(37.1)	
										
Race	Han	166(100.0)	103(100.0)	-	269(77.5)	-	-	121(63.4)	148(60.7)	0.257
	Kazakh	-	-	78(100.0)	78(22.5)	-		36(18.8)	42(17.2)	
	Caucasion	-	-	-	-	41(46.6)		18(9.4)	23(9.4)	
	Black	-	-	-	-	8(9.1)		5(2.6)	3(1.2)	
	Japanese	-	-	-	-	39(44.3)		11(5.8)	28(11.5)	
										
Anatomic location*	Upper third	20(18.3)	-	1(1.6)	-	-	-	14(17.9)	7(7.5)	0.115
	Middle third	71(65.1)	-	33(53.2)	-	-		45(57.7)	59(63.4)	
	Lower third	18(16.5)	-	28(45.2)	-	-		19(24.4)	27(29.0)	
										
TNM stage	I	7(4.2)	21(20.4)	0(0.0)	28(8.1)	31(35.2)	<0.001	20(10.5)	39(16.0)	0.204
	IIa	84(50.6)	26(25.2)	34(43.6)	144(41.5)	28(31.8)		77(40.3)	95(38.9)	
	IIb	35(21.1)	37(36.0)	34(43.6)	106(30.5)	12(13.7)		59(30.9)	59(24.2)	
	III	40(24.1)	19(18.4)	10(12.8)	69(19.9)	17(19.3)		35(18.3)	51(20.9)	

†P values for the differences between China and US were calculated with the Pearson Chi-square test.

††P values for the differences between HPV(+) and HPV(-) groups were calculated with the Pearson Chi-square test.

‡Tobacco was classified as any record of use (ever) or no record of use (never). Information on tobacco was missing in four cases.

‡‡Alchol was classified as any record of use (ever) or no record of use (never). Information on tobacco was missing in eight cases.

*Part of the data was not available, and statistic analyses were based on available data.

### Quality control

DNA preparation, PCR setup, and PCR product detection were carried out in separated spaces with specimens moving through the laboratory in one direction only. Environmental contamination was monitored consistently before experiments, and decontamination was carried out on a regular basis. To monitor potential contamination during DNA preparation, one mouse liver tissue sample was inserted after each batch of 16 esophageal cancer tissues and processed with these cancer tissues. The human globin gene and HPV L1 were tested in these controls to confirm absence of contamination during DNA preparation.

In each 96-well PCR reaction plate the following controls were included: four mouse liver DNA samples processed together with the human cancer specimens as a DNA preparation control; three controls without a DNA template; two sets of HPV16 plasmid DNA, each containing 100, 10, 1 and 0.1 copies admixed with 100 ng of human genome DNA; and one positive control containing 100 ng of ESCC (esophageal squamous cell carcinoma) DNA mixed together from several tumors, which had previously tested positive for HPV L1.

Assay results were interpreted and used for this study only when controls met the following criteria: 1. all seven negative controls were negative; 2. ESCC DNA was positive; and 3. at least one set of plasmid controls was positive and the intensity of the positive signal corresponded with the input copy number. In cases where any negative control was positive, testing was repeated.

### DNA preparation

DNA from 88 US samples was extracted in the NCI laboratory (Maryland, USA) using the FlexiGene kit (Qiagen, Valencia, CA). DNA from all other samples was prepared using the H.Q. & Q. Tissue DNA Kit (U-GENE BIOTECHNOLOGY CO., LTD, Anhui, China). DNA was diluted to 35 ng/μl, and 3 μl (~100 ng) of DNA was used for each of the various PCR analyses.

### HPV L1 detection

A set of primers, SPF1/GP6+, which amplify an L1 fragment of approximately 184 bp were used. These primer sets were reported previously to be highly sensitive [19]. The polymerase chain reaction was carried out under the following conditions. Qiagen Hot Start Taq DNA polymerase mixture was used with 2 mM MgCl_2_, and 16 pmol of each primer. Enzyme activation was carried out at 95°C for 15 minutes, followed by 45 amplification cycles at 95°C for 40 seconds, 49°C for 50 seconds, 72°C for 30 seconds, and a final extension at 72°C for 5 minutes.

### HPV genotype determination and detection

To determine the HPV genotypes in samples for each region from different geographic areas, L1 positive samples were mixed in equal molar ratios for each region and cloned into a TA vector. Forty-eight colonies from each batched cloning were sequenced.

Type-specific detection of HPV was performed with type-specific PCR primers (Table 2) for genotypes detected from batched cloning. PCR was performed at 95°C for 15 minutes, followed by 40 amplification cycles at 95°C for 40 seconds, 57°C for 40 seconds, 72°C for 40 seconds, and a final extension at 72°C for 5 minutes.

**Table 2:** HPV type-specific primers and sizes of PCR-amplified fragments†

HPV type	Primer direction††	Primer sequence	Gene	Size of PCR fragment (bp)
6	F	5'-CTGTTTCGAGGCGGCTATC-3'	E6	323
	R	5'-TGGAGGTTGCAGGTCTAAT-3'		
16	F	5'-ATGACTTTGCTTTTCGGGATTTAT-3'	E6E7	335
	R	5'-GCATGATTACAGCTGGGTTTCTC-3'		
18	F	5'-AACCGAGCACGACAGGAACG-3'	E7	368
	R	5'-GGATGCACACCACGGACACA-3'		
26	F	5'-TGACCTACGCTGCTACGAACAA-3'	E7	294
	R	5'-CCCGCCCCTCCTCATTT-3'		
45	F	5'-ACGACCCTACAAGCTACCAGATTT-3'	E6	454
	R	5'-TTGCTATACTTGTGTTTCCCTACG-3'		
56	F	5'-TGGGGTGCTGGAGACAAACA-3'	E7	271
	R	5'-CTGCACCACAAACTTACACTCACA-3'		
57	F	5'-ATACCCGAAATTGTTGACCT-3'	E7	182
	R	5'-TGCTCCAGATGCCTTATGT-3'		
58	F	5'-CCAGGACGCAGAGGAGAAACC-3'	E6	387
	R	5'-CGACCCGAAATATTATGAAACCTT-3'		

†PCR: abbreviation for polymerase chain reaction.

††F, forward; R, reverse.

### Sequencing analysis

PCR products were purified using a PCR clean-up gel extraction column (MACHEREY-NAGEL GmbH & Co, Düren, Germany) according to the manufacturer's instructions and were directly sequenced using a capillary sequencer (ABI Prism 3100).

### Statistical analysis

Univariate comparisons of rates were performed using the chi-square test. Unconditional multivariate logistic regression was used to compare the rates of multiple type infections and HPV detection rates among different regions adjusting for potential confounders. All statistical analysis was done using SAS 9.1.3. *P *values of less than 0.05 were considered to be statistically significant.

## Results

### Overall HPV prevalence according to region

Of 435 of esophageal cancer samples, 244 (56.1%) were HPV L1 positive. Stratifying by region, HPV L1 was detected in 86 (51.8%) of the Anyang samples, 62 (60.2%) of the Shantou samples, 42 (53.8%) of the Xinjiang samples, and 54 (61.4%) of the US samples. There were no significant differences among the detection rates in the samples from the three areas of China (*P *= 0.131) (Table 3). In addition, in comparing samples from China (54.8%) and the US (61.4%), no significant difference was observed (*P *= 0.522) (Table 3).

**Table 3:** HPV detection rate and multiple infection rate by region*

	HPV L1(+)						
			
Region	Unclassified No. (%)	Single infection No. (%)	Multiple infection No. (%)	***P***	Total No. (%)	HPV L1 (-) No. (%)	*P*
Anyang (N = 166)	20(23.3)	57(66.3)	9(10.5)	0.963†	86(51.8)	80(48.2)	0.131‡
Shantou (N = 103)	4(6.5)	43(69.4)	15(24.2)		62(60.2)	41(39.8)	
Xinjiang (N = 78)	6(14.3)	30(71.4)	6(14.3)		42(53.8)	36(46.2)	
China (N = 347)	30(15.8)	130(68.4)	30(15.8)	0.834††	190(54.8)	157(45.2)	0.522‡‡
US (N = 88)	1(1.9)	40(74.1)	13(24.1)		54(61.4)	34(38.6)	
Total (N = 435)	31(12.7)	170(69.7)	43(17.6)		244(56.1)	191(43.9)	

*Multiple infection was determined by type specific E6 or E7 PCR.

†P value for the rates of multiple type infections among three areas of China was calculated by unconditional logistic regression adjusted for age, gender, tobacco, alcohol and pathological stage.

††P value for the rates of multiple type infections between China and US was calculated by unconditional logistic regression adjusted for age, gender, tobacco, alcohol and pathological stage.

‡P value for HPV detection rates among three areas of China was calculated by unconditional logistic regression adjusted for age, gender, tobacco, alcohol and pathological stage.

‡‡P value for HPV detection rates between China and US was calculated by unconditional logistic regression adjusted for age, gender, tobacco, alcohol and pathological stage.

### Type specific HPV prevalence according to region and epidemiological characteristics

Using the batched cloning method, a total of eight genotypes including HPV6, 16, 18, 26, 45, 56, 57, and 58 were found. Type-specific primer (Table 2) derived PCR was then carried out on L1 positive samples. The most prevalent genotype was HPV16 which was detected in 96 (49.2%) of the China samples, of which 29 were from Anyang, 48 were from Shantou, 19 were from Xinjiang, and in 39 (58.2%) of the US samples. The difference of detection rate of HPV16 between China and US was statistically significant (*P *= 0.003). HPV57 was the second most prevalent genotype, and was found in 46 (23.6%) of the China samples, of which 27 were from Anyang, 8 were from Shantou, 11 were from Xinjiang, and in 14 (20.9%) of the US samples. In addition, HPV26 was also common both in China and US with a prevalence of 9.2% in China and 13.4% in the US. HPV18 was found in three of the four study areas with a detection rate of 14.5% in Anyang, 8.1% in Shantou, and 7.5% in the US. HPV6, 45, 56, and 58 were much less common. Three cases (4.0%) from Anyang were HPV45 positive. Nine (20.0%) and 4 (8.9%) cases from Xinjiang were HPV6 and HPV56 positive respectively (Table 4). And all these differences were not reaching the significant standard. In addition, we further compared the detection rates of each genotype by epidemiological characteristics, including age, gender, smoking and alcohol use. However, no statistical differences were found (data not shown).

**Table 4:** Type-specific HPV infection distribution by region

		China					
				
HPV type	Total No. (%)	Anyang No. (%)	Shantou No. (%)	Xinjiang No. (%)	Total No. (%)	US No. (%)	P*
HPV-6	9(3.4)	0 (0.0)	0 (0.0)	9(20.0)	9(4.6)	0 (0.0)	0.268
HPV-16	135(51.5)	29(38.1)	48(64.9)	19(42.2)	96(49.2)	39(58.2)	0.003
HPV-18	22(8.4)	11(14.5)	6(8.1)	0 (0.0)	17(8.7)	5(7.5)	0.979
HPV-26	27(10.3)	6(7.9)	10(13.5)	2(4.4)	18(9.2)	9(13.4)	0.080
HPV-45	3(1.1)	3(4.0)	0 (0.0)	0 (0.0)	3(1.5)	0 (0.0)	1.000
HPV-56	4(1.5)	0 (0.0)	0 (0.0)	4(8.9)	4(2.1)	0 (0.0)	0.587
HPV-57	60(22.9)	27(35.5)	8(10.8)	11(24.4)	46(23.6)	14(20.9)	0.519
HPV-58	2(0.8)	0 (0.0)	2(2.7)	0 (0.0)	2(1.0)	0 (0.0)	1.000
Total	262(100.0)	76(100.0)	74(100.0)	45(100.0)	195(100.0)	67(100.0)	

*P values for type-specific HPV detection rates according to region in which subject live were calculated with the Pearson Chi-square test or Fisher's exact test.

Among L1 positive samples, 43 (17.6%) were co-infected with more than one HPV type. Multiple HPV genotypes were found in 30 (15.8%) cases from China, of which 9 were from Anyang, 15 were from Shantou, 6 were from Xinjiang, and in 13 cases (24.1%) from the US among the L1 positive samples. In addition, no significant differences in multiple infection rates were observed among three areas of China (*P *= 0.963) or between China and the US (*P *= 0.834).

### Effect of tumor type and tumor site on HPV prevalence

HPV infection in esophageal adenocarcinomas and squamous cell carcinomas was compared among the samples from the US. L1 was detected in 18 (52.9%) of esophageal adenocarcinomas and 36 (66.7%) of esophageal squamous cell carcinomas (*P *= 0.200). The detection rates in these 2 types of carcinomas therefore showed no statistically significant difference. Tumor site data was available only for squamous cell carcinomas from the Anyang and Xinjiang regions, and evaluation of the association between anatomic location and HPV prevalence was carried out in these cases. L1 was detected in 7 (33.3%) of carcinomas located in the upper third of esophagus, 59 (56.7%) in the middle third and 27 (58.7%) in the lower third. No statistically significant relationship between HPV infection and tumor site was found in this study (P = 0.115).

### Impact of potential risk factors on HPV prevalence

HPV infection was further analyzed on the basis of several potential risk factors (Table 5). However, the L1 positive rate did not show any statistically significant association with sex, age, race, or TNM stage. In contrast, HPV positivity was associated with alcohol use (OR = 1.69, 95% CI = 1.01-2.83). Samples were stratified into cases from China and from the US, and upon repetition of the above analysis, identical results were observed in the Chinese cases. However, HPV positives were not linked with alcohol use in the US samples (data not shown).

**Table 5:** Risk of esophageal cancer associated with HPV infection according to gender, age, tobacco and alcohol use, and tumor stage

		HPV(-) No.(%)	HPV(+) No.(%)	Unadjusted OR (95% CI)		Adjusted OR (95% CI)*	
Age	≤60	121(63.4)	141(57.8)	1.00		1.00	
	> 60	70(36.6)	103(42.2)	1.26	(0.86-1.86)	1.17	(0.78-1.75)
							
Gender	Female	63(33.0)	65(26.6)	1.00		1.00	
	Male	128(67.0)	179(73.4)	1.36	(0.90-2.05)	1.06	(0.64-1.75)
							
Tobacco†	No	104(55.3)	111(45.7)	1.00		1.00	
	Yes	84(44.7)	132(54.3)	1.47‡	(1.00-2.16)	1.08	(0.64-1.80)
							
Alcohol††	No	143(76.5)	151(62.9)	1.00		1.00	
	Yes	44(23.5)	89(37.1)	1.92‡‡	(1.25-2.94)	1.69‡	(1.01-2.83)
							
TNM stage	I	20(10.5)	39(16.0)	1.00		1.00	
	IIa	77(40.3)	95(38.9)	0.63	(0.34-1.17)	0.68	(0.36-1.28)
	IIb	59(30.9)	59(24.2)	0.51	(0.27-0.98)	0.58	(0.30-1.14)
	III	35(18.3)	51(20.9)	0.75	(0.38-1.49)	0.74	(0.36-1.50)

*Age, gender, smoking, tobacco use, alcohol use and TNM stage were included in the multivariate logistic regression model.

†Information on tobacco was missing in four cases.

††Information on alcohol was missing in eight cases.

‡P<0.05.

‡‡P<0.01.

## Discussion

Over the past twenty years, many reports regarding HPV infection in esophageal cancer have been published, and the reported HPV detection rate in these tumors has ranged from 0 to 67% [15,17,20-23]. To explain these marked differences in the reported detection rate, sampling methods, demographic and ethnic factors, disease status, and sensitivity of detection methods have been cited as potential causes of inconsistency. HPV was detected infrequently in esophageal carcinomas collected from western countries, whereas it was more frequently detected in esophageal cancers from regions of high HPV incidence, particularly in China and South Africa. It therefore has been proposed that HPV infection may play a role in esophageal carcinogenesis only in high incidence regions [24,25]. In contrast to previous reports, our results demonstrated the prevalence of HPV infection in esophageal cancer is independent of geographic area and ethnicity, suggesting HPV infection is commonly associated with esophageal cancer and raising the possibility that it may play a role in the etiology of esophageal carcinoma.

The differences in prevalence of HPV in esophageal carcinoma in this study and previous reported prevalence may have resulted in part from use of different analytic strategies. We recently demonstrated two ways in which HPV infection in the esophagus differs from the infection observed in cancers of the uterine cervix (data not shown). First, real time PCR revealed that the HPV load in esophageal specimens was at least two orders of magnitude lower than that in cervical cancer. In addition, HPV genotypes found in esophageal specimens were different from those found in cervical cancers. Based on these observations, we proposed in a previous study that the low HPV copy number might therefore result in a falsely low detection rate in esophageal cancer. To provide evidence for this line of reasoning, a series of HPV 16 positive cervical cancer specimens were employed to test a mimic esophageal cancer model. The copy number of HPV/cell in these cervical cancers was first determined using real time PCR. The samples were then diluted to 0.1-0.01/cell which was similar to the average copy number in esophageal cancers and tested for L1. Of 24 diluted samples, 6 were negative in the first round of PCR. An additional round of PCR was carried out, and all six samples which were negative in the first PCR analysis were positive, indicating that repeat testing may decrease the false negative rate in samples with low HPV copy numbers. On this basis, an analytic strategy for HPV detection in esophageal cancers was formulated.

Briefly, L1 detection was performed twice in each esophageal sample. L1 positivity in either test was counted as positive for calculation of the detection rate. A batched TA cloning assay was used to evaluate the HPV genotypes in the sample population. Utilizing this approach, HPV57 which is not common in cervical cancers was identifiable in esophageal specimens. Genotype specific E6 or E7 PCR was performed to determine the genotypes in L1 positives.

It is of interest to note that HPV57 presented as the second most common genotype and accounted for 22.9% of L1 positive esophageal cancer tissues from all four areas under investigation. HPV57 was originally identified in benign, premalignant, and malignant lesions of the nasal cavity [26]. The signature pattern of HPV57 E6 and E7 oncogenes was closely related to HPV16 and 18 oncogenes. NIH 3T3 cells expressing HPV57 exhibited morphological transformation indicating that the activity of HPV57 in vitro is comparable to other high-risk HPV types [27]. The role of HPV57 in esophageal carcinogenesis thus warrants further investigation.

The associations between HPV infection and age, gender, tumor TNM stage, tobacco smoking and alcohol use were analyzed. Of these factors only alcohol use was associated with HPV positive esophageal cancers as compared with HPV negative subjects. However, there was no statistical evidence of such relationship when the analysis was limited to US samples only, but the possibility that the limited US sample size might have contributed to this result cannot be excluded. Alternatively, as samples from the US included both esophageal squamous cell carcinomas and adenocarcinomas, it is possible that these two types of cancers may arise from different carcinogenic pathways and thus confound the data. As such, further investigation with a larger sample size should be carried out to further evaluate the effect of alcohol use on HPV infection in the esophagus.

We recognize that there are several limitations in this study, one of which is selection bias. As a hospital-based study, these cancer patients were all recruited from local hospitals and as a result the population from which we obtained these samples cannot be precisely defined. Patient self selection of a hospital for treatment may have also introduced selection bias. These factors may limit the sample's representativeness and capacity for inference. Another limitation is that some crucial information which would potentially allow broader conclusions was not obtained for this study (for example, anatomic location of the tumor in the US samples). In this study we demonstrated for the first time that HPV DNA is both relatively common in esophageal cancer, and is independent of geographic region and ethnicity of tested subjects. In addition, HPV infection appears to be independent of all previously identified risk factors for esophageal cancer except alcohol use.

## Conclusions

This observed consistency of the prevalence of HPV infection in esophageal carcinoma from different geographic areas may warrant further investigation with larger numbers of subjects over additional areas. However, the findings here serve to raise the possibility that HPV plays a role in the etiology of esophageal carcinoma.

## Competing interests

The authors declare that they have no competing interests.

## Authors' contributions

XW and XT carried out HPV detection and typing. FL performed the statistical analysis. YZ, and MS participated in HPV detection. DC participated in the design of statistical analysis. CL, ZW, XS, QZ, DZ, ZS, FL and CH participated in sample preparation, data validation and study coordination. HC and YK conceived the study, and participated in its design and coordination and prepared the manuscript. All authors read and approved the final manuscript.

## Pre-publication history

The pre-publication history for this paper can be accessed here:

http://www.biomedcentral.com/1471-2407/10/19/prepub
